# Human MiniPromoters for ocular-rAAV expression in ON bipolar, cone, corneal, endothelial, Müller glial, and PAX6 cells

**DOI:** 10.1038/s41434-021-00227-z

**Published:** 2021-02-02

**Authors:** Andrea J. Korecki, Jorge L. Cueva-Vargas, Oriol Fornes, Jessica Agostinone, Rachelle A. Farkas, Jack W. Hickmott, Siu Ling Lam, Anthony Mathelier, Michelle Zhou, Wyeth W. Wasserman, Adriana Di Polo, Elizabeth M. Simpson

**Affiliations:** 1 Centre for Molecular Medicine and Therapeutics at BC Children’s Hospital, University of British Columbia, Vancouver, BC Canada; 2 Department of Neuroscience, University of Montreal Hospital Research Centre, University of Montreal, Montreal, QC Canada; 3 Department of Medical Genetics, University of British Columbia, Vancouver, BC Canada

**Keywords:** Gene therapy, Gene expression analysis, Neurological disorders

## Abstract

Small and cell-type restricted promoters are important tools for basic and preclinical research, and clinical delivery of gene therapies. In clinical gene therapy, ophthalmic trials have been leading the field, with over 50% of ocular clinical trials using promoters that restrict expression based on cell type. Here, 19 human DNA MiniPromoters were bioinformatically designed for rAAV, tested by neonatal intravenous delivery in mouse, and successful MiniPromoters went on to be tested by intravitreal, subretinal, intrastromal, and/or intravenous delivery in adult mouse. We present promoter development as an overview for each cell type, but only show results in detail for the recommended MiniPromoters: Ple265 and Ple341 (*PCP2*) ON bipolar, Ple349 (*PDE6H*) cone, Ple253 (*PITX3*) corneal stroma, Ple32 (*CLDN5*) endothelial cells of the blood–retina barrier, Ple316 (*NR2E1*) Müller glia, and Ple331 (*PAX6*) PAX6 positive. Overall, we present a resource of new, redesigned, and improved MiniPromoters for ocular gene therapy that range in size from 784 to 2484 bp, and from weaker, equal, or stronger in strength relative to the ubiquitous control promoter smCBA. All MiniPromoters will be useful for therapies involving small regulatory RNA and DNA, and proteins ranging from 517 to 1084 amino acids, representing 62.9–90.2% of human proteins.

## Introduction

Small and cell-type restricted promoters are important tools for basic and preclinical research, and clinical delivery of gene therapies. Recent basic research examples include restricted promoters to study the blood retinal barrier (BRB) [1], understand critical periods in photoreceptor development [2], and validate novel recombinant adeno associated virus (rAAV) capsids [3, 4]. In preclinical research, restricted promoters have been used to rescue numerous animal models of ocular disease [5–10]. In clinical gene therapy, ophthalmic trials have been leading the field [11], with over 50% of rAAV-based ocular therapies using promoters that restrict expression based on cell type (www.clinicaltrials.gov) [8, 12–14]. Promoters that restrict expression have already demonstrated increased efficacy [6, 15, 16], and safety by limiting any given therapy to the target cells, thereby reducing unwanted off-target effects [15, 17–19]. In clinical applications, restricted promoters may come from either the therapeutic gene or, if the therapeutic gene shows abnormal expression with disease, from an unrelated gene with the appropriate expression pattern for the therapy [11–14].

Despite the number of restricted promoters currently available, more are needed. rAAV is the vector of choice for gene therapy of the eye, and with a limited laboratory packaging size of ~4.9 kb [20], additional restricted promoters of varying length, and thus ability to deliver different kinds of therapies, will be necessary. Additionally, promoters of varying strength will also be required. For many therapeutic strategies endogenous-like expression may be optimal, thereby avoiding the toxicity of over expression [21], but for applications such as optogenetics, strong expression is a must [22–24]. Promoters that function in the developmental context, as well as in adults, will be needed as delivery to children for life-long expression is expanded [25, 26]. Finally, numerous therapeutic target cell types are underserved by the current collection of promoters.

Promoters for six ocular cell types were developed in this work: ON bipolar, cone, corneal, endothelial, Müller glia, and paired box 6 (*PAX6*). ON bipolar neurons transmit signals from the photoreceptors to the ganglion cells, and are an important target for various forms of complete congenital stationary night blindness [6], melanoma-associated retinopathy [27, 28], and optogenetic strategies [16, 29, 30]. Dysfunction of cone photoreceptors, which are responsible for color vision, can lead to various disorders including: achromatopsia, blue-cone monochromatism, and Stargardt disease [31, 32]. The corneal stroma is essential for the maintenance of clear vision and normal visual function, and diseases of the cornea include corneal neovascularization, genetic anterior and stromal dystrophies, and monogenic lysosomal storage disorders [33]. The BRB is necessary in establishing and maintaining a suitable environment for retinal function, and impairment of the barrier can lead to the accumulation of blood-borne proteins and other potentially toxic solutes [34], and is directly associated with both diabetic retinopathy and wet age-related macular degeneration [35]. Müller glia span the entire retina and provide essential molecules to the retinal neurons, are important targets for macular telangiectasia type 2 [36] and *retinitis pigmentosa* [8], and may be able to deliver widely a neuroprotective therapeutic to the retina [37]. The *PAX6* gene is expressed in a variety of tissues including the central nervous system and pancreas, but is best known as the essential transcription factor for ocular development [38–41], and involvement in the ocular disorder aniridia [42, 43]. A major challenge in the development of PAX6 gene therapy is that expression of the endogenous protein is complex, and inappropriate locations and levels of PAX6 could be detrimental [44–47].

Building on this previous work of ours and others, we present our current strategy for MiniPromoter (minimal human promoter element(s) designed for expression in restricted cell types) design and testing, and the resulting resource. We used human DNA sequence, which may decrease our success during promoter development in mice, but increase the likelihood of success when a MiniPromoter is transitioned to primates [48] and the clinic. We chose the starting genes based on the literature and single-cell transcriptomics data [49, 50]. We have included the native transcription start site for its immediately flanking regulator value. We then bioinformatically identified candidate regulatory element(s) and placed them in a non-native configuration 5′ of the start site. If a “first-generation” MiniPromoter was successful, we typically designed subsequent generations based on that “source”, applying three design strategies to improve the promoter. (1) “Redesigned” MiniPromoters contained both regions of the source MiniPromoter, but also new genomic sequence. (2) “Cut-down” MiniPromoters had sequence removed from the source MiniPromoter. (3) “Base pair modified” MiniPromoters had mutations introduced to the source promoter, but size was not altered. MiniPromoters were tested in rAAV9, which was chosen for its ability to cross the neonatal blood–brain barrier and minimal capsid-based cellular specificity [51], allowing any observed restriction of expression to reside primarily with the promoter. Successful MiniPromoters were tested using a variety of delivery methods including direct intravitreal and subretinal delivery to the adult eye, intrastromal delivery into the adult cornea, and adult intravenous delivery. We present promoter development as an overview for each cell type, but only show results in detail for the “recommended” MiniPromoter(s).

## Materials and methods

### MiniPromoter design

The bioinformatics designs for MiniPromoters that were “Unaltered” (Table 1) have been described previously [52–55]. The bioinformatics designs for MiniPromoters that were “Redesigned”, “Cut down”, or “Base pair modified” (Table 1) are described in the “Results”. The bioinformatics designs for MiniPromoters that were “New” (Table 1) were done as follows: for the chosen genes, the identification of regulatory regions (RRs) was limited to within neighboring-gene boundaries or regulatory confinements by topologically associating domains as identified by Hi-C data [56]. It then relied on the integration of multiple sources of evidence: transcribed promoter and enhancer regions (identified through cap analysis of gene expression (CAGE) [57, 58]) and genomic run-on sequence data [59], predicted promoter and enhancer regions (ChromHMM and Segway [60, 61]), chromatin accessibility (DNase I data [62]), transcription factor-bound regions and histone modifications (identified by ChIP-seq data [62]), and multi-species conservation [63]. The final MiniPromoter design sequence was obtained by placing one promoter region at the 3′ end and adding additional enhancer regions as allowed by the desired final size of the construct.

**Table 1:** Summary of MiniPromoters characterized in rAAV9 Ple#-intron-EmGFP-WPRE.

Cells chosen for	Gene	Ple	MiniP size (bp)	MiniP plasmid (pEMS)	rAAV plasmid (pEMS)	Source (PMID)	Design type	Expression related to	Figure(s)
ON bipolar	*PCP2*	155^a^	1652	1936	2115^b^	KI mouse (24761428, 30765420), rAAV (26310623, 27164903)	Unaltered	Gene	1A
ON bipolar	*PCP2*	265^c^	986	1942^b^	2214^b^	KI mouse (30765420)	Unaltered	Gene	1A–D and S2A
ON bipolar	*PCP2*	318	986	2234	2240	Ple265	Base pair modified	Negative	1A
ON bipolar	*PCP2*	341^c^	784	2267^b^	2271^b^	Ple265	Cut down	Gene	1A
Cone	*GNGT2*	347	1197	2277	2282	Genome	New	Gene	2A–D and S2B
Cone	*PDE6H*	348	2025	2278	2283	Genome	New	Gene	3A
Cone	*PDE6H*	349^c^	2005	2279^b^	2284^b^	Genome	New	Gene	2C, 3A–C, and S2C
Cornea	*PITX3*	253^c^	2484	1920^b^	2230^b^	KI mouse (30765420), rAAV (27164903)	Unaltered	Design Source	4A–D and S2D
Endothelial BRB	*CLDN5*	32^c^	1670	1503^b^	2181^b^	KI mouse (20807748, 24761428)	Unaltered	Gene	5A–E and S2E
Endothelial BRB	*CLDN5*	261	2963	1938^b^	2218	rAAV (27164903, 31323276)	Unaltered	Gene	5A
Endothelial BRB	*CLDN5*	338	2567	2172	2268	Ple261	Redesigned	Gene	5A
Endothelial BRB	*CLDN5*	339	1973	2173	2269	Ple261	Redesigned	Gene	5A
Endothelial BRB	*CLDN5*	340	2700	2174	2270	Ple261	Redesigned	Gene	5A
Müller glia	*NR2E1*	264	3026	1941^b^	2215	KI mouse (30765420), rAAV (27164903)	Unaltered	Gene	6A
Müller glia	*NR2E1*	315	1996	2231	2237	Ple264	Cut down	Gene	6A
Müller glia	*NR2E1*	316^c^	1691	2232^b^	2238^b^	Ple264	Redesigned	Gene	6A–D and S2F
PAX6 positive	*PAX6*	255^a^	2049	1928^b^	2044^b^	rAAV (27556059)	Unaltered	Gene	7A
PAX6 positive	*PAX6*	259^a^	2087	1932^b^	2048^b^	rAAV (27556059)	Unaltered	Gene	7A
PAX6 positive	*PAX6*	328	2148	2163	2257	Ple259 with Ple255 (27556059)	Redesigned	Gene	7A
PAX6 positive	*PAX6*	329	2513	2164	2258	Ple259 with Ple255 (27556059)	Redesigned	Gene	7A
PAX6 positive	*PAX6*	330	1982	2165	2259	Genome	New	Gene	7A
PAX6 positive	*PAX6*	331^c^	1982	2166^b^	2260^b^	Genome	New	Gene	7A–E and S2G

*BRB* blood–retina barrier, *pEMS* plasmid Elizabeth M. Simpson, *Ple* Pleiades MiniPromoter.

^a^Listed for comparison, but not recharacterized in this work.

^b^Plasmids available at Addgene (www.addgene.org).

^c^Recommended MiniPromoter.

### Cloning and virus production

MiniPromoter design sequences were ordered for direct DNA synthesis (GenScript, Inc., Piscataway, NJ), or isolated from previous Pleiades Promoter plasmids [52–55], and cloned into the multiple cloning site (*Avr*II, *Fse*I, *Mlu*I, and *Asc*I) of our “plug and play” rAAV2 backbone plasmid version 2 (pEMS2131) [54]. This genome plasmid has been previously described [48, 54], and includes: an intron, *Not*I flanked emerald green fluorescent protein (EmGFP), *Asi*SI flanked woodchuck hepatitis virus posttranscriptional regulatory element (WPRE), and SV40 polyA. Plasmids were propagated in *E. coli* SURE cells (Agilent Technologies, Santa Clara, CA). DNA was prepared by Spin MiniPrep Kit (QIAgen, Germantown, MD) and plasmids were confirmed free of rearrangements by *Ahd*I digest, ITRs verified by *Sma*I digest, and cloning sites verified by sequencing. The ubiquitous smCBA promoter plasmid was pEMS2143, and MiniPromoter plasmids are listed in Table 1. Confirmed plasmids were sent to the University of Pennsylvania Vector Core (Philadelphia, PA) for large-scale DNA amplification using the EndoFree Plasmid Mega Kit (QIAgen). Quality control on the plasmid preparation was done by *Sma*I, *Pvu*II, and *Sna*BI digests, and confirmed plasmids were packaged into rAAV9 capsid with titer determined by droplet digital PCR.

### Intravenous injection and histology in neonatal mice

All experimental mice were B6129F1 hybrids of both sexes, produced as the first-generation by crossing C57BL/6J (JAX Stock No: 000664) to 129S1/SvImJ (JAX Stock No: 002448). Postnatal day (P) 0 and P4 pup injections were into the superficial temporal vein; a 31-gauge needle on a 0.33 cc syringe (320440, BD, Franklin Lakes, NJ) is inserted under the skin ~1–2 mm parallel to the vessel, and then advanced into the vein, 50 µl of virus at a titer of 1 × 10^13^ genome copies/ml (GC/ml) (or 3.33 × 10^12^ GC/ml for smCBA and *PAX6* MiniPromoters) in phosphate buffer saline (PBS) and 0.001% pluronic acid (together, PBS + P) was slowly injected. Following injection, the pups were tattooed for identification and returned to their home cage with the dam. At 1 week for central nervous system tissues (eye, brain, spinal cord) for *CLDN5* MiniPromoters, or at 4 weeks for all other tissues and MiniPromoters, mice were given a lethal dose of avertin (MilliporeSigma, Burlington, MA), perfused transcardially with 4% paraformaldehyde, the eyes, brain, spinal cord, liver, heart, and pancreas were dissected, and tissues were postfixed for 2 h at 4 °C. There was no randomization of mice.

Tissues were cryoprotected with 25% sucrose overnight at 4 °C and then embedded in optimal cutting temperature compound (Tissue-Tek, Sakura Finetek, Torrance, CA) on dry ice and 20 μm cryosections were directly mounted onto slides. Sections were stained as previously described [48] using primary antibody anti-GFP (GFP-1020, Aves Labs Inc., Tigard, OR; 1:500), secondary antibody Alexa Fluor 488 (Life Technologies, Carlsbad, CA; 1:1000), and Hoechst (Sigma-Aldrich Cat# 881405, St. Louis, MO; 1:1000). A minimum of two animals were studied for each injection time point for each MiniPromoter (i.e. a minimum of four animals per MiniPromoter), the consistency of MiniPromoter constructs across animals was validated in a previous publication [54]. Unique expression patterns of the promoters were determined by microscope and image analysis. The images shown are typically from mice that had the most successful injections and thus the full dose of rAAV, and therefore the greatest likelihood to observe off-target expression if present. Fluorescent images were standardly taken using an Olympus BX61 motorized microscope (Olympus, Shinjuku, Japan).

Mice were housed in the pathogen-free Centre for Molecular Medicine and Therapeutics facility on a 7 a.m.–8 p.m. light cycle, 20 ± 2 °C with 50 ± 5% relative humidity, with food and water ad libitum. All procedures involving mice were in accordance with the guidelines of the Canadian Council on Animal Care for the Use of Experimental Animals and approved by the UBC Animal Care Committee (Protocols A14-0294, A14-0295, A17-0204, A17-0205).

### Subretinal, intravitreal, intrastromal, and intravenous injection and histology in adult mice

Delivery route was chosen based on the target cell type. All injections were into C57BL/6NCrl mice of both sexes (19–22 g, Charles River International, Saint Constant, Quebec). The viruses were diluted in PBS + P. For the subretinal injections, 1 µl of virus at a titer of 1 × 10^13^ GC/ml were injected into the subretinal space using Wiretrol ll capillary micropipettes (Drummond Scientific Co., Broomall, PA). The tip of the glass micropipette (diameter ~100 µm) was inserted parallel to the eye wall into the subretinal space, between the retinal pigment epithelium and the retina. The use of the micropipette allows self-sealing of the puncture site without reflux of the solution and minimal injury. For intravitreal injections, 2 µl of virus at a titer of 1 × 10^13^ GC/ml were injected into the vitreous chamber. The injection was performed using a 10 µl Hamilton syringe (Hamilton Company, Reno, NV) adapted with a 32-gauge glass microneedle. The tip of the needle was inserted into the superior hemisphere of the eye at a ∼45° angle through the sclera into the vitreous body to avoid retinal detachment or injury to eye structures. All injections were performed in the right eye and treated with one drop of Vigamox (Alcon Laboratories, Fort Worth, TX) to minimize the risk of infection. For intrastromal injections, 1 µl of virus at a titer of 1 × 10^13^ GC/ml were injected into the stromal space using Wiretrol ll capillary micropipettes. The tip of the glass micropipette was inserted parallel to the eye wall into the stromal space. For intravenous injections, 200 µl of virus at a titer of 1 × 10^12^ GC/ml were injected into the tail vein using a 28-gauge insulin syringe (329461, BD). There was no randomization of mice.

Four weeks after virus delivery, anesthetized animals were perfused transcardially with 4% paraformaldehyde, and both eyes were rapidly dissected and postfixed for 2–4 h at 4 °C. For retinal sections and whole mounts, the cornea and lens were removed and the eyecup was incubated overnight in 30% sucrose. For corneal sections, the globe was dissected out and incubated overnight in 30% sucrose. For sections, the tissue was then embedded in optimal cutting temperature compound (Tissue-Tek) and retinal or corneal cross sections (16 µm) were generated and collected onto slides (Fisherbrand, Pittsburgh, PA). Sections were stained as previously described [48] using primary antibodies: anti-GFP (A-11122, Life Technologies), anti-PCP2 (sc-68356, Santa Cruz Biotechnologies, Dallas, TX); anti-opsin (AB5407, MilliporeSigma); anti-CD31 (551262, BD Biosciences, San Jose, CA); anti-CRALBP (MA1-813, Thermo Fisher Scientific, Waltham, MA); anti-RBPMS (PhosphoSolutions, Aurora, CO); and anti-PAX6 (901301, BioLegend, San Diego, CA), and secondary antibodies: anti-rabbit or mouse IgG Alexa Fluor 488 (Life Technologies); anti-mouse IgG Alexa Fluor 594 (Life Technologies). Sections were washed and mounted in SlowFade Diamond Antifade mountant with DAPI (Life Technologies) for visualization with an Axio Imager.M2 Microscope (Zeiss, Oberkochen, Germany).

Mice were housed in the Centre de Recherche du Centre Hospitalier de l’Université de Montréal (CRCHUM) facility on a 12-h light/dark cycle, with food and water ad libitum. All procedures involving mice were in accordance with the guidelines of the Canadian Council on Animal Care for the Use of Experimental Animals and approved by the CRCHUM Animal Care Committee (Protocol N15009ADPs).

### Quantification of EmGFP epifluorescence intensity in adult mice

Four mice were used per group, and individual co-labeled- or morphology-identified EmGFP-positive cells were manually traced. The area and the integrated density (the summation of the pixels value in the traced area) were measured with ImageJ software (http://rsbweb.nih.gov/ij/). Areas with no apparent fluorescence were also traced and used as background. For an epifluorescence comparison, we used tissues transduced with a virus carrying the ubiquitous smCBA promoter. Importantly, images were taken at the same exposure time with no adjustments made. Analyses were done at settings where there was a dynamic range of EmGFP expression in the target cells from weak to strong. Analysis and statistics were performed using the Graphpad Instat software (Version 5.01, GraphPad Software Inc., San Diego, CA) using a Student’s *t* test or where multiple MiniPromoters are compared using ANOVA, post hoc analysis. All results are reported as the mean ± standard deviation. Analysis was not performed blinded to the MiniPromoter.

### Image processing

Images were processed using ImageJ (http://rsbweb.nih.gov/ij/), Photoshop, and Illustrator (Adobe, San Jose, CA). Brightness, contrast, and color balancing adjustments were made in ImageJ (neonatal injected) or Photoshop (adult injected) where necessary to improve visibility.

### Availability of data and materials

Plasmids for cloning, control and recommended MiniPromoters, and corresponding virus genomes are available to the research community through the nonprofit distributor Addgene (Cambridge, MA) (www.addgene.org). For MiniPromoter plasmid numbers please see Table 1. pEMS2131, rAAV2 backbone plasmid version 2; pEMS2075, control smCBA promoter plasmid; pEMS2143, control smCBA virus genome plasmid.

## Results

### Five delivery methods showed robust and ubiquitous expression of rAAV9 smCBA-EmGFP

Neonatal and adult intravenous delivery, direct intravitreal and subretinal delivery to the adult eye, and intrastromal delivery into the adult cornea were all tested using the rAAV9 capsid and the ubiquitous smCBA promoter driving EmGFP. A positive result ensured we would have access to that cell type for MiniPromoter development. A negative result did not distinguish between lack of capsid transduction (rAAV9 does not transduce all cell types), or lack of promoter expression (smCBA is ubiquitous but not universal). The former mechanism would prevent development of a MiniPromoter for that cell type using this capsid, but the latter mechanism does not preclude the cell type from MiniPromoter development using this system.

Testing by intravenous delivery in neonatal mouse allowed us to examine not only expression in the eye, but peripheral tissues as well. Since some eye disorders are syndromes with additional negative impacts on a variety of organ systems, these data will enable strategizing promoter use not only for ocular, but also systemic treatment of disease. Only MiniPromoters that showed desirable expression patterns following neonatal intravenous delivery were studied further. rAAV9 smCBA-EmGFP was injected at either postnatal day (P) 0 or P4 to evaluate the different cell types of the eye, as it has previously been shown that the development of the BRB occurring during this window results in expression primarily in different cell types [64]. As previously published, P0 injection results primarily in ganglion, amacrine, horizontal, photoreceptor, and corneal stroma cell expression; and P4 injection results in primarily ganglion, amacrine, bipolar, and Müller glia expression (Fig. S1A). Regardless of the date of injection, positive staining was seen in all brain regions (including both neuronal and glial cells), and throughout the spinal cord, liver, heart, and pancreas, which were the peripheral tissues studied (Fig. S1B).

Two delivery methods are commonly used to directly target the adult retina: subretinal and intravitreal. Subretinal injections have been widely used for rAAV gene therapy in research, clinical trials, and approved therapeutics, although it is considered to be more traumatic due to the need to penetrate the retina and induce temporary retinal detachment [65, 66]. In comparison, intravitreal injection of rAAV is technically easier to perform and less invasive, and is therefore more desirable for the clinic [66, 67]. We tested both methods when evaluating MiniPromoters designed to target the retina. Our results with rAAV9 smCBA-EmGFP demonstrated expression in various cell types in the mouse retina, regardless of injection route (Fig. S1C, D).

Intrastromal injections have been used in the clinic to directly treat the adult cornea [68, 69], and for preclinical rAAV gene therapy [70, 71]. Intrastromal injections in mice have been demonstrated to transduce all layers of the cornea, as well as reach the limbal stem cells [72]. We therefore used intrastromal injection to evaluate MiniPromoters designed to target the cornea. Our results with rAAV9 smCBA-EmGFP demonstrated expression in the injected cells of the corneal stroma (Fig. S1E).

rAAV9 does not transduce tissues in adult mice as efficiently as neonates when delivered intravenously, due to reduced ability to cross the adult BBB [51]. We therefore limited use of adult intravenous delivery to MiniPromoters designed to target the BRB and BBB. Our results with rAAV9 smCBA-EmGFP demonstrated that despite the lowered efficiency of rAAV9 in adult mice, expression was observed in various retinal cells (Fig. S1F).

### ON bipolar MiniPromoter primary recommendation is Ple265 (*PCP2*, 986 bp)

In brief, our data support the recommendation of Ple265 (*PCP2*, 986 bp) as a MiniPromoter for the ON bipolar cells for its versatility giving robust-specific expression by neonatal intravenous, and direct subretinal injection. If size is the most critical determinant, Ple341 (*PCP2*, 784 bp) is also excellent for intravenous delivery, with less robust subretinal results.

We evaluated three MiniPromoters for ON bipolar cells, all based on the Purkinje cell protein gene (*PCP2*, aka *L7*). *PCP2* was chosen as it is well recognized that, in addition to the brain expression for which it is named, it has specificity to ON bipolar retinal cells [73–75].

**Fig. 1:**
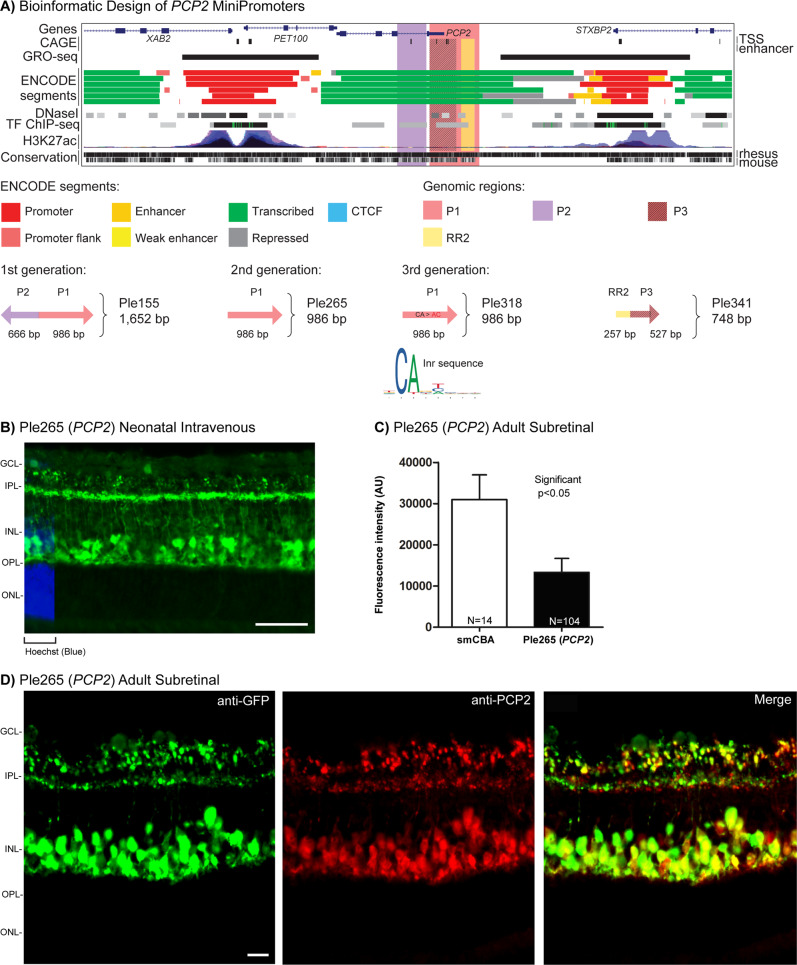
Development of *PCP2* MiniPromoters for ON bipolar cells. **A** Bioinformatic design of first-generation Ple155, second-generation Ple265, base pair modification to produce third-generation Ple318, and cutdown to produce third-generation Ple341. ENCODE segments that potentially regulate the expression of the *PCP2* gene are highlighted horizontally. Vertically highlighted genomic regions correspond to their color-matched segments included in the MiniPromoter designs and are numbered as promoter(s) (P) and regulatory region(s) (RR). **B** Postnatal day 4 intravenous injection of Ple265-EmGFP, harvested 4 weeks later, led to robust expression in the inner nuclear layer (INL). Scale bar, 100 µm. (For ubiquitous smCBA promoter see Fig. S1A.) **C** Adult subretinal injection of Ple265-EmGFP, harvested 4 weeks later, was quantified for epifluorescence intensity in PCP2-positive cells, and showed significantly lower (~1/2×) expression compared to smCBA (*p* < 0.05). (For smCBA see Fig. S1C.) **D** Adult subretinal injection of Ple265-EmGFP, harvested 4 weeks later, led to robust expression in ON bipolar cells, as indicated by co-staining with the ON bipolar cell marker PCP2. Scale bar, 10 µm. (For smCBA see Fig. S1C.) EmGFP emerald green fluorescent protein, GCL ganglion cell layer, gen generation, Inr initiator element, IPL inner plexiform layer, N number of cells counted, ONL outer nuclear layer, OPL outer plexiform layer, TF transcription factor, TSS transcription start site. Green, anti-GFP; blue, Hoechst; red, anti-PCP2; yellow, merge.

A first-generation MiniPromoter Ple155 (*PCP2*, 1,652 bp) (Table 1 and Fig. 1A), previously showed robust expression in both ON bipolar and Purkinje cells in the cerebellum, when used in knock-in mice, and rAAV2(quadY-F + T-V) or rAAV9 viruses [6, 53–55]. Ple155 was also used to ameliorate a mouse model of complete stationary night blindness [6], and to study bipolar cell fates in the developing retina [2]. A second-generation cut down of Ple155, MiniPromoter Ple265 (*PCP2*, 986 bp) (Table 1 and Fig. 1A), was bioinformatically designed to maintain expression in ON bipolar cells, while eliminating Purkinje cell expression, and rendering the promoter smaller [55]. Previous testing of Ple265 in knock-in mice confirmed the maintenance of ON bipolar expression, but due to high background expression in the brain we were unable to confirm the elimination of the Purkinje cell expression [55]. Here, we retested Ple265 for ON bipolar expression and elimination of brain expression, using rAAV.

A third-generation base pair modified MiniPromoter Ple318 (*PCP2*, 986 bp), based on Ple265 (*PCP2*), was designed to eliminate bioinformatically predicted testis expression while enhancing the ON bipolar expression (Table 1 and Fig. 1A). FANTOM5 CAGE data [57, 58] showed three transcription start sites in the Ple265 promoter, two of them being testis specific. To eliminate this predicted testis expression, the sequences corresponding to initiator sites were disrupted by mutating the CAs to ACs. These changes undesirably created a potential *NEUROD2* binding site, which was subsequently destroyed by base pair modification. To enhance bipolar expression, sequences corresponding to four binding sites for Sebox/Vsx1 transcription factors were destroyed since the literature shows they express in ON bipolar cells and may functions as repressors [76–80]. In parallel, another third-generation cut down Ple341 (*PCP2*, 784 bp), also based on Ple265, was designed to maintain the ON bipolar expression pattern in an even smaller promoter (Table 1 and Fig. 1A). Based on multi-species conservation [63], a 200 bp RR and 527 bp promoter were cut out of Ple265 and put together to create Ple341.

By neonatal intravenous delivery at P4 (the optimal injection time point for bipolar cells [64]), Ple265 (*PCP2*) expression was robust and restricted to the inner nuclear layer (INL) of the retina, with a pattern indicative of bipolar cells (Fig. 1B). Expression was absent in the brain, spinal cord, and heart, but was observed at a low level in the liver (Fig. S2A). Importantly, these data allowed us to demonstrate that this design successfully eliminated the expression in Purkinje cells. At P0, expression was again observed in the INL and at a low level in the ganglion cell layer (GCL).

Ple318 (*PCP2*) delivered at P4 resulted in a significant reduction of expression in the INL, as well as the addition of expression in the GCL. By altering the transcription factor binding site and transcription start site, Ple318 significantly reduced, instead of strengthened, ON bipolar expression. Thus, this MiniPromoter was not studied further. Ple341 (*PCP2*) delivered at P4 resulted in no detectable difference in the bipolar cell like expression pattern in the INL when compared to Ple265 (*PCP2*). In addition, expression was also absent in the brain, spinal cord, and heart, but was observed at a low level in the liver and pancreas.

The two most INL robust MiniPromoters (indicative of bipolar cells) by intravenous delivery, Ple265 (*PCP2*) and Ple341 (*PCP2*) went on to be characterized in the adult mouse eye by both subretinal (Fig. 1C, D) and intravitreal injection. Ple265 resulted in robust and restricted expression to the INL by subretinal delivery (Fig. 1D), although not by intravitreal delivery. Ple341 resulted in less robust expression restricted to the INL by both subretinal and intravitreal delivery.

Quantification was done for the most robust-specific MiniPromoter Ple265 (*PCP2*) by adult direct subretinal injection. Quantification of EmGFP epifluorescence intensity in individual PCP2-positive ON bipolar cells transduced with the same dose of virus, showed that Ple265-driven expression was reduced by ~1/2× relative to the ubiquitous smCBA promoter (*p* < 0.05) (Fig. 1C). Co-labeling with anti-PCP2, an ON bipolar cell marker, not only confirmed expression in ON bipolar cells, but the endogenous-like expression pattern of the MiniPromoter (Fig. 1D).

### Cone MiniPromoter recommendation is Ple349 (*PDE6H*, 2005 bp)

In brief, our data support the recommendation of Ple349 (*PDE6H*, 2005 bp) as a MiniPromoter for cone cells for its versatility giving strong and robust-specific expression by neonatal intravenous, and direct subretinal and intravitreal injection.

We evaluated three MiniPromoters for the cones, based on the genes: guanine nucleotide binding protein (G protein), gamma transducing activity polypeptide 2 (*GNGT2*) and phosphodiesterase 6H, cGMP-specific, cone, gamma (*PDE6H*). Drop-seq data were used as the primary criterion for gene selection, with *PDE6H* being the top result, and *GNGT2* the third [50]. Criteria for final selection included: strong and enriched expression in the retina with clear transcription start site prediction from FANTOM CAGE data [57, 58], small gene size with low structural complexity [81], high sequence conservation from the UCSC genome browser, and expression conservation between mouse and human from the literature [82–84].

RRs were classified into promoters (regions overlapping a transcription start site, as indicated by CAGE data) or enhancers, and the most promising regions were placed into MiniPromoter constructs. We used 2–2.5 kb as our target MiniPromoter size, with the aim to include enough regulatory sequence to have a successful promoter with restricted expression in the initial design, while being minimal enough for rAAV delivery of therapeutic RNAs and small protein-coding sequences. This work resulted in the design of three first-generation MiniPromoters: Ple347 (*GNGT2*, 1197 bp), Ple348 (*PDE6H*, 2025 bp), and Ple349 (*PDE6H*, 2005 bp) (Table 1).

**Fig. 2:**
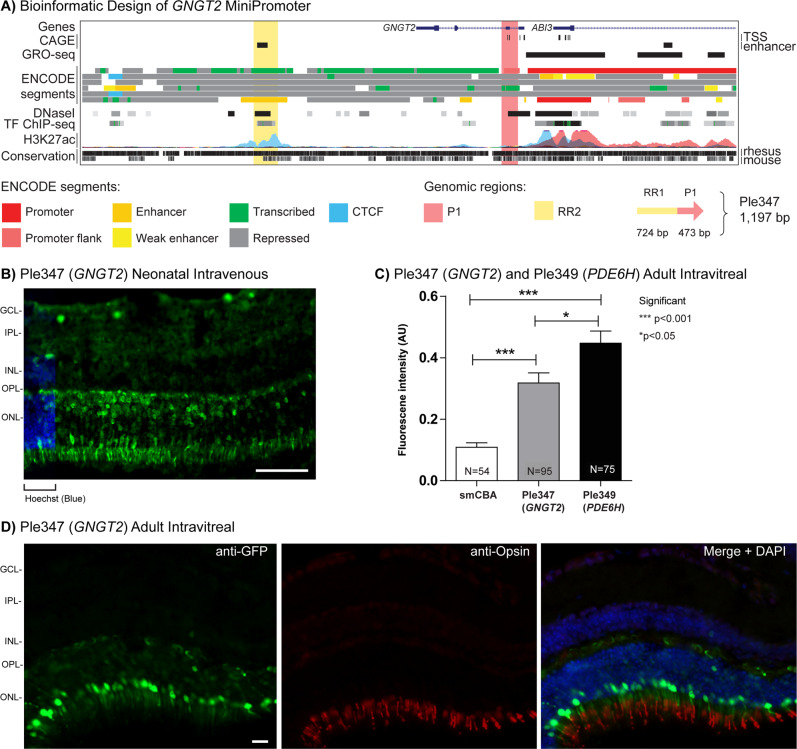
Development of *GNGT2* MiniPromoters for cone cells. **A** Bioinformatic design of Ple347. ENCODE segments that potentially regulate the expression of the *GNGT2* gene are highlighted horizontally. Vertically highlighted genomic regions correspond to their color-matched segments included in the MiniPromoter design and are numbered as promoter(s) (P) and regulatory region(s) (RR). **B** Postnatal day 0 intravenous injection of Ple347-EmGFP, harvested 4 weeks later, led to robust expression in the outer nuclear layer (ONL). Scale bar, 100 µm. (For ubiquitous smCBA promoter see Fig. S1A). **C** Adult intravitreal injection of Ple347-EmGP and Ple349 (*PDE6H*)-EmGFP, harvested 4 weeks later, was quantified for epifluorescence intensity in opsin-positive cells, and showed that Ple347 (*GNGT2*) expression was significantly increased (~3×) compared to smCBA (*p* < 0.001). In addition, Ple349 (*PDE6H*) expression was significantly increased compared to both smCBA (~4×) (*p* < 0.001) and Ple347 (~1.5×) (*p* < 0.05). (For smCBA see Fig. S1D.) **D** Adult intravitreal injection of Ple347-EmGFP, harvested 4 weeks later, led to robust expression in cone cells, as indicated by co-staining with the cone cell marker opsin where EmGFP inner segments align to anti-opsin outer segments. Scale bar, 20 µm. (For smCBA see Fig. S1D.) EmGFP emerald green fluorescent protein, GCL ganglion cell layer, INL inner nuclear layer, IPL inner plexiform layer, N number of cells counted, OPL outer plexiform layer, TF transcription factor, TSS transcription start site. Green, anti-GFP; blue, Hoechst; red, anti-opsin.

By neonatal intravenous delivery at P0 (the optimal injection time point for photoreceptors), Ple347 (*GNGT2*) expression was robust in the outer nuclear layer (ONL) (Fig. 2B). Expression was absent in the brain, spinal cord, and heart, and observed at a low level in the pancreas, and a moderate level in the liver (Fig. S2B). After injection at P4, expression was observed in the ONL, but also in the GCL and INL.

Ple347 (*GNGT2*) went on to be characterized in the adult mouse eye, and showed robust and restricted expression to the ONL by both subretinal and intravitreal injection, the latter more stringently demonstrating the specificity of this MiniPromoter because it must cross the entire retina and only express when it reaches the cones (Fig. 2D). Quantification was done for the most robust cone-specific MiniPromoters for each gene by adult direct intravitreal injection: Ple347 (*GNGT2*) and Ple349 (*PDE6H*). Quantification of EmGFP epifluorescence intensity in individual opsin-positive cone cells transduced with the same dose of virus, showed that Ple347-driven expression was increased (~3×) relative to the ubiquitous smCBA promoter (*p* < 0.001) (Fig. 2C). In addition, Ple349-driven expression was increased from both the smCBA promoter (~4×) (*p* < 0.001), and Ple347 (~1.5×) (*p* < 0.05) (Fig. 2C). Co-labeling with anti-opsin, a cone cell marker, confirmed expression of Ple347 in cone cells, where EmGFP is primarily localized to the inner segment and aligns to anti-opsin in the outer segment (Fig. 2D).

**Fig. 3:**
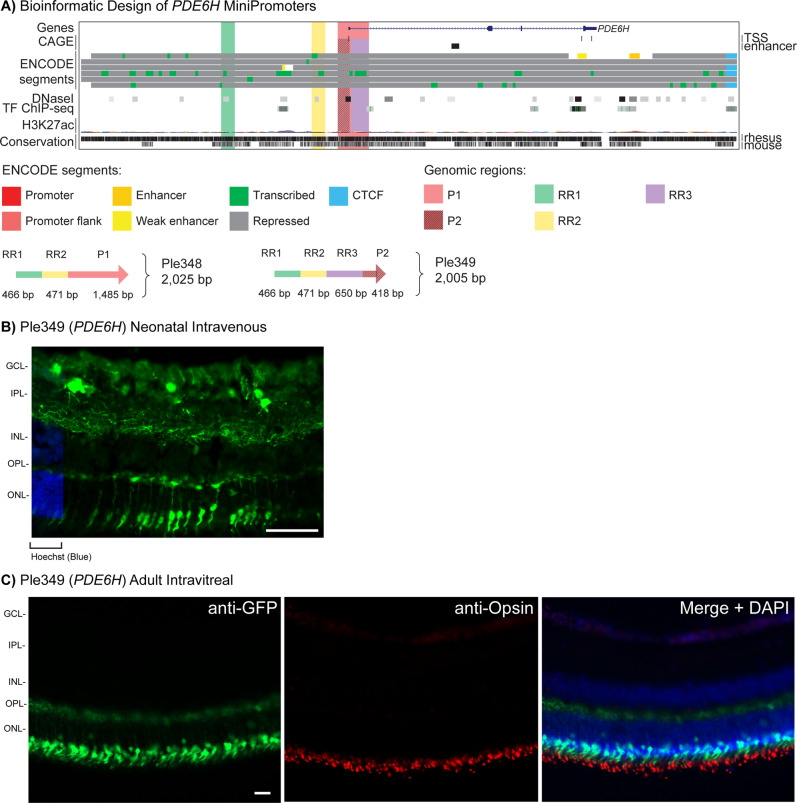
Development of *PDE6H* MiniPromoters for cone cells. **A** Bioinformatic design of Ple348 and Ple349. ENCODE segments that potentially regulate the expression of the *PDE6H* gene are highlighted horizontally. Vertically highlighted genomic regions correspond to their color-matched segments included in the MiniPromoter design and are numbered as promoter(s) (P) and regulatory region(s) (RR). **B** Postnatal day 0 intravenous injection of Ple349-EmGFP, harvested 4 weeks later, led to robust expression in the outer nuclear layer (ONL). Scale bar, 100 µm. (For ubiquitous smCBA promoter see Fig. S1A.) **C** Adult intravitreal injection of Ple349-EmGFP, harvested 4 weeks later, led to robust expression in cone cells, as indicated by co-staining with the cone cell marker opsin, where EmGFP inner segments align to anti-opsin outer segments. Scale bar, 20 µm. (For smCBA see Fig. S1D.) EmGFP emerald green fluorescent protein, GCL ganglion cell layer, INL inner nuclear layer, IPL inner plexiform layer, OPL outer plexiform layer, TF transcription factor, TSS transcription start site. Green, anti-GFP; blue, Hoechst; red, anti-opsin.

First-generation MiniPromoters Ple348 (*PDE6H*) and Ple349 (*PDE6H*) consist of two different promoter regions, two shared RRs, and one additional RR only found in Ple349 (Table 1 and Fig. 3A). By neonatal intravenous delivery at P0, Ple348 resulted in expression in the ONL and the GCL, at P4 expression was observed in the GCL and INL. Thus, this MiniPromoter was not studied further. In contrast, Ple349 expression was robust and enriched in the ONL (Fig. 3B). Expression of EmGFP in the processes was observed in the brain stem and the dorsal region of the spinal cord; expression was also observed at a low level in the liver and pancreas, and there was no expression observed in the heart (Fig. S2C). At P4, expression was observed at a low level in the GCL and INL.

Ple349 (*PDE6H*) went on to be characterized in the adult mouse eye, and showed robust and restricted expression to the ONL by both subretinal and again more stringently by intravitreal delivery (Fig. 3C). Co-labeling with anti-opsin, a cone cell marker, confirmed expression in cone cells (Fig. 3C). Overall, this is the recommended MiniPromoter of the three designs for cone cells.

### Corneal stroma MiniPromoter recommendation is Ple253 (*PITX3*, 2484 bp)

In brief, our data support the recommendation of Ple253 (*PITX3*, 2484 bp) as a MiniPromoter for the corneal stroma for its enriched stromal expression by neonatal intravenous, and direct intrastromal injection.

**Fig. 4:**
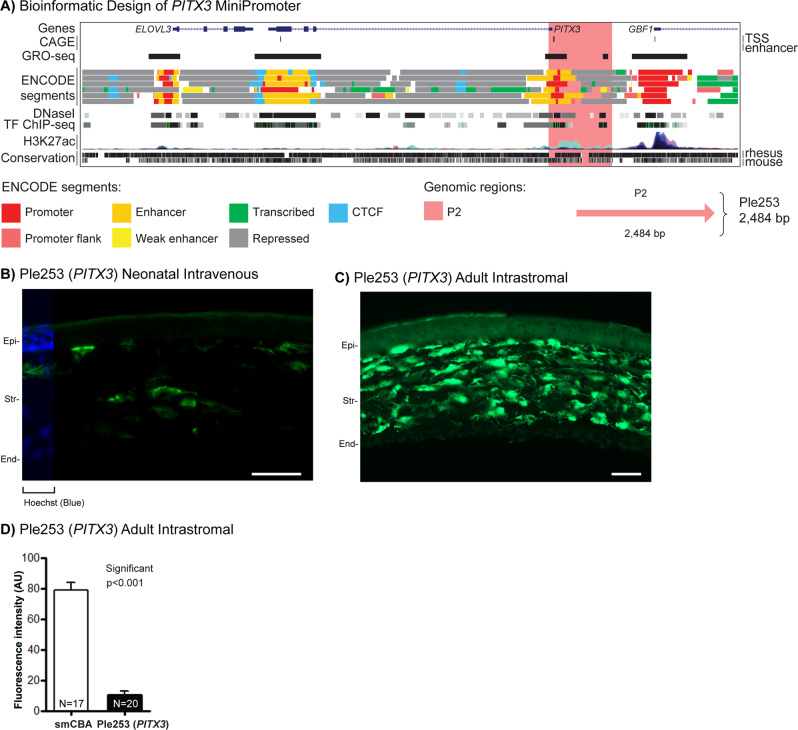
Development of *PITX3* MiniPromoters for corneal stroma cells. **A** Bioinformatic design of Ple253. ENCODE segments that potentially regulate the expression of the *PITX3* gene are highlighted horizontally. Vertically highlighted genomic regions correspond to their color-matched segments included in the MiniPromoter design and are numbered as promoter (P). **B** Postnatal day 0 intravenous injection of Ple253-EmGFP, harvested 4 weeks later, led to robust expression in the stromal layer (Str) of the cornea, as determined from Hoechst staining and cell morphology. Scale bar, 100 µm. (For ubiquitous smCBA promoter see Fig. S1A). **C** Adult intrastromal injection of Ple253-EmGFP, harvested 4 weeks later, led to robust expression in the stromal layer, as determined by cell morphology. Scale bar, 50 µm. (For smCBA see Fig. S1E.) **D** Adult intrastromal injection of Ple253-EmGFP, harvested 4 weeks later, was quantified for epifluorescence intensity in stromal cells, determined by cell morphology, was and showed significantly lower (~1/5×) expression compared to smCBA (*p* < 0.001). (For smCBA see Fig. S1E.) EmGFP emerald green fluorescent protein, End endothelial layer, Epi epithelial layer, N number of cells counted, TF transcription factor, TSS transcription start site. Green, anti-GFP; blue, Hoechst.

Paired-like homeodomain transcription factor 3 (*PITX3*) was originally selected for expression in the substantia nigra [85, 86], although our initial testing found MiniPromoter Ple253 (*PITX3*) to be negative in the brain (Table 1 and Fig. 4A) [54]. We did, however, demonstrate that Ple253 showed enriched expression in the corneal stroma when intravenously delivered with rAAV driving improved cre recombinase [54]. However, it had never previously been tested with a direct reporter such as EmGFP or by direct adult injection, thus its applicability as a promoter for therapy was limited.

By neonatal intravenous delivery at P0 (the optimal injection time point for the cornea [48]), Ple253 (*PITX3*) expression was robust and enriched for the stromal layer of the cornea (Fig. 4B). Expression was absent in the brain (including the substantia nigra), spinal cord, and heart, and was observed at a moderate level in the liver and the pancreas (Fig. S2D). By intravenous delivery at both P0 and P4, Ple253 expression was observed at a moderate level in both the GCL and INL (Fig. S2D).

Ple253 (*PITX3*) went on to be characterized in the adult mouse eye by intrastromal injection. Ple253 resulted in robust expression specifically in the stromal layer, as determined by location and cell morphology (Fig. 4C). Quantification of EmGFP epifluorescence intensity in individual corneal stroma cells, as determined by cell morphology, and transduced with the same dose of virus, showed that Ple253-driven expression was reduced by ~1/5× relative to the ubiquitous smCBA promoter (*p* < 0.001) (Fig. 4D).

### Endothelial blood–retina barrier MiniPromoter recommendation is Ple32 (*CLDN5*, 1670 bp)

In brief, our data support the recommendation of Ple32 (*CLDN5*, 1670 bp) as a MiniPromoter for the endothelial cells of the BRB for its versatility giving robust-specific expression by neonatal and adult intravenous delivery, and its relative size compared to our other *CLDN5* MiniPromoter designs.

We evaluated five MiniPromoters for the endothelial BRB, all based on the Claudin 5 (*CLDN5*) gene. CLDN5 is an integral membrane protein and component of tight junctions [87], which develop between endothelial cells of the blood vessels in the central nervous system, and play a key role in establishing the BRB and BBB.

**Fig. 5:**
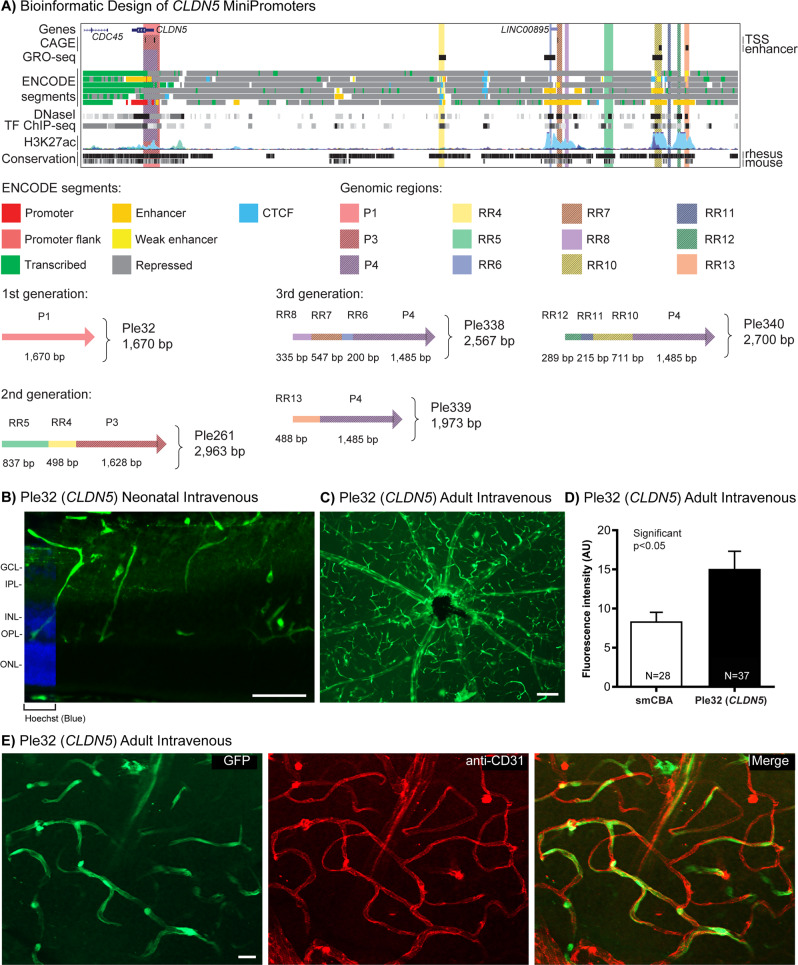
Development of *CLDN5* MiniPromoters for endothelial cells of the blood–retina barrier. **A** Bioinformatic design of first-generation Ple32, and redesign to produce second- and third-generation Ple261, Ple338, Ple339, and Ple340. ENCODE segments that potentially regulate the expression of the *CLDN5* gene are highlighted horizontally. Vertically highlighted genomic regions correspond to their color-matched segments included in the MiniPromoter designs and are numbered as promoter(s) (P) and regulatory region(s) (RR). **B** Postnatal day 4 intravenous injection of Ple32-EmGFP, harvested 1 week later, led to robust expression in the blood vessels, as determined by morphology. Scale bar, 100 µm. (For ubiquitous smCBA promoter see Fig. S1A.) **C** Adult intravenous injection of Ple32-EmGFP, harvested 4 weeks later, and visualized by whole mount retina, led to robust expression in the blood vessels, as determined by morphology. Scale bar, 100 µm (For smCBA see Fig. S1F.) **D** Adult intravenous injection of Ple32-EmGFP, harvested 4 weeks later, was quantified for epifluorescence intensity in CD31-positive cells, and showed significantly higher (~2×) expression compared to smCBA (*p* < 0.01). (For smCBA see Fig. S1F). **E** Adult intravenous injection of Ple32-EmGFP, harvested 4 weeks later, led to robust expression in the endothelial cells of the blood–retina barrier, as indicated by co-staining with the endothelial cell marker CD31. Scale bar, 20 µm. (For smCBA see Fig. S1F.) EmGFP emerald green fluorescent protein, GCL ganglion cell layer, INL inner nuclear layer, IPL inner plexiform layer, N number of cells counted, ONL outer nuclear layer, OPL outer plexiform layer, TF transcription factor, TSS transcription start site. Green, anti-GFP; blue, Hoechst; red, anti-CD31; yellow, merge.

We have previously demonstrated that first-generation MiniPromoter Ple32 (*CLDN5*, 1670 bp) (Table 1 and Fig. 5A) shows restricted expression in endothelial cells of the BRB and BBB in knock-in mice [52, 53], but it had never been tested using rAAV. Previously used in rAAV was a substantially larger second-generation MiniPromoter Ple261 (*CLDN5*, 2963 bp) (Table 1 and Fig. 5A) [1, 3, 4, 53]. Due to both the size, and off-target expression of Ple261 in the brain by intravenous neonatal delivery (see below), we decided to re-evaluate the first-generation Ple32 but now in rAAV, and design three third-generation MiniPromoters to evaluate new RRs.

Three third-generation MiniPromoters were developed to reduce off-target expression: Ple338 (*CLDN5*, 2567 bp), Ple339 (*CLDN5*, 1973 bp), and Ple340 (*CLDN5*, 2700 bp) (Table 1 and Fig. 5A). The three redesigns follow the bioinformatic methods outlined above, all using the same promoter region (cut down from Ple32 and Ple261), with the addition of several new RRs from the genome.

By neonatal intravenous delivery at P4, with an early 1 week harvest at P11 to capture EmGFP expression that could be lost due to cell proliferation during blood vessel development [88], Ple32 (*CLDN5*) expression was robust and enriched for blood vessels in the retina (Fig. 5B). Expression was also observed in blood vessels of the brain and spinal cord (Fig. S2E). At the standard 4-week time point, low levels of expression were observed in the heart and pancreas, and a moderate level of expression was observed in the liver (Fig. S2E). At the P11 time point, liver expression was higher.

Second- and third-generation redesigns Ple261 (*CLDN5*), Ple338 (*CLDN5*), Ple339 (*CLDN5*), and Ple340 (*CLDN5*), delivered intravenously as described for Ple32, resulted in a higher level of off-target expression in the brain and the peripheral tissues. Thus, these MiniPromoters were not studied further.

Typically, AAV9 is not used intravenously in adult mice because at this age it does not cross the BBB efficiently [51]. However, it was exactly this property that allowed us to ask if the virus was able to reach the target endothelia cells of the blood vessels despite not efficiently crossing the barrier. Using the most robust-specific blood vessel MiniPromoter by neonatal intravenous delivery Ple32 (*CLDN5*), we found adult delivery also resulted in robust and restricted expression to the three vascular layers of the eye (Fig. 5C–E) and the blood vessels of the brain (Fig. S2E).

Quantification of EmGFP epifluorescence intensity in individual CD31-positive endothelial cells transduced with the same dose of virus, showed that Ple32 (*CLDN5*)-driven expression was increased by ~2× relative to the ubiquitous smCBA promoter (*p* < 0.05) (Fig. 5D). Co-labeling with anti-CD31, an endothelial cell marker, confirmed that the blood vessel expression was in the target endothelial cells that create the barrier (Fig. 5E), and that the virus need not cross the barrier for promoter expression in these cells.

### Müller glia MiniPromoter recommendation is Ple316 (*NR2E1*, 1691 bp)

In brief, our data support the recommendation of Ple316 (*NR2E1*, 1691 bp) as a MiniPromoter for the Müller glia for its versatility giving robust-specific expression by neonatal intravenous, and adult intravitreal delivery.

We evaluated three MiniPromoters for the Müller glia, all based on the Nuclear receptor subfamily 2, group E, member 1 (*NR2E1*) gene. NR2E1 is a transcriptional regulator that has been demonstrated to play a critical role in brain and eye development [89–92]. *NR2E1* was chosen for the Müller glia of the retina based on the literature [90, 91, 93].

**Fig. 6:**
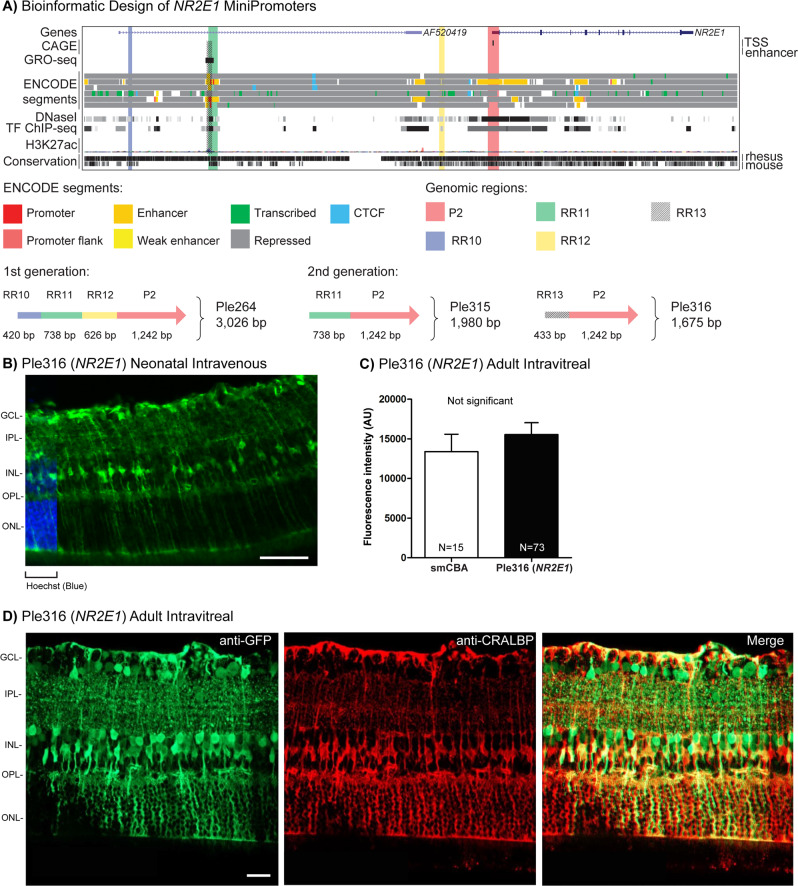
Development of *NR2E1* MiniPromoters for Müller glia cells. **A** Bioinformatic design of first-generation Ple264, cutdown to produce second-generation Ple315, and redesign to produce second-generation Ple316. ENCODE segments that potentially regulate the expression of the *NR2E1* gene are highlighted horizontally. Vertically highlighted genomic regions correspond to their color-matched segments included in the MiniPromoter designs and are numbered as promoter(s) (P) and regulatory region(s) (RR). **B** Postnatal day 4 intravenous injection of Ple316-EmGFP, harvested 4 weeks later, led to robust expression with cell bodies in the inner nuclear layer (INL) and processes that span the retina. Scale bar, 100 µm. (For ubiquitous smCBA promoter see Fig. S1A.) **C** Adult intravitreal injection of Ple316-EmGFP, harvested 4 weeks later, was quantified for epifluorescence intensity in CRALBP-positive cells, and showed no significant difference when compared to smCBA. (For smCBA see Fig. S1D). **D** Adult intravitreal injection of Ple316-EmGFP, harvested 4 weeks later, led to robust expression in the Müller glia cells, as indicated by co-staining with Müller glia cell marker CRALBP. Scale bar, 20 µm. (For smCBA see Fig. S1D.) EmGFP emerald green fluorescent protein, GCL ganglion cell layer, IPL inner plexiform layer, N number of cells counted, ONL outer nuclear layer, OPL outer plexiform layer, TF transcription factor, TSS transcription start site. Green, anti-GFP; blue, Hoechst; red, anti-CRALBP; yellow, merge.

We have previously demonstrated that first-generation MiniPromoter Ple264 (*NR2E1*, 3026 bp) (Table 1 and Fig. 6A) showed restricted expression in the Müller glia of the retina when delivered with rAAV [54] and knock-in mice [55], but it had never been tested by adult direct injection into the eye. Based on this previous work, we did not expect to capture the endogenous brain expression of *NR2E1* with the second-generation MiniPromoters based on Ple264.

Second-generation MiniPromoters were designed to reduce size and increase specificity: Ple315 (*NR2E1* 1980 bp) and Ple316 (*NR2E1*, 1675 bp) (Table 1 and Fig. 6A). Cut down Ple315 contains the promoter region from Ple264 (*NR2E1*), along with only one RR (RR11), an enhancer. Redesign Ple316 also contains the promoter region from Ple264, along with a redesigned RR (RR13). FANTOM5 analysis revealed the enhancer was only partially captured by RR11, and this new RR13 was therefore redesigned to capture the entire enhancer.

By neonatal intravenous delivery at P4 (the optimal injection time point for Müller glia), Ple316 (*NR2E1*) expression was robust and restricted to cell bodies in the INL and radial processes that span the retina, morphology indicative of Müller glia (Fig. 6B). Expression was absent in the spinal cord, heart, and pancreas, and was observed at a low level in the glia and neurons of the brain, and a low level in the liver (Fig. S2F). After P0 intravenous injection, expression was observed at a low level in non-glial cells in the GCL and INL. In contrast, first-generation Ple264 (*NR2E1*) resulted in higher off-target expression in the GCL, while cut down Ple315 (*NR2E1*) resulted in both higher off-target expression in the GCL as well as lower expression in the Müller glia. Ple264 was studied further for comparison with redesign Ple316, but Ple315 was not studied further.

Ple316 (*NR2E1*) by intravitreal injection again resulted in robust and enriched expression with cell bodies in the INL and processes that span the retina, a defining feature of Müller glia (Fig. 6C, D). Subretinal delivery resulted in only modest expression of the Müller glia. Ple264 (*NR2E1*) resulted in robust expression in the GCL and few cells with a morphology indicative of Müller glia, both by adult intravitreal and subretinal delivery, thus was not considered Müller glia specific.

Quantification was done for Ple316 (*NR2E1*), the most robust Müller glia-specific MiniPromoter by direct intravitreal delivery in adult mice. Quantification of EmGFP epifluorescence intensity in individual CRALBP-positive Müller glia cells transduced with the same dose of virus, showed that Ple316-driven expression was ~1× and not significantly different relative to the ubiquitous smCBA promoter (Fig. 6C). Co-labeling with anti-CRALBP, a Müller glia marker, confirmed expression in Müller glia cells (Fig. 6D). Co-labeling also revealed the main off-target expression of Ple316 to be the amacrine cells.

### PAX6-positive cell MiniPromoter recommendation is Ple331 (*PAX6* SIMO, 1982 bp)

In brief, our data support the recommendation of Ple331 (*PAX6* SIMO, 1982 bp) as a MiniPromoter for the PAX6-positive cells for its versatility giving robust-specific expression by neonatal intravenous, and adult subretinal delivery. Importantly, of all the *PAX6* MiniPromoters evaluated, Ple331 was the only one that expressed in all four PAX6-positive cell types of the retina: ganglion, amacrine, horizontal, and Müller glia.

**Fig. 7:**
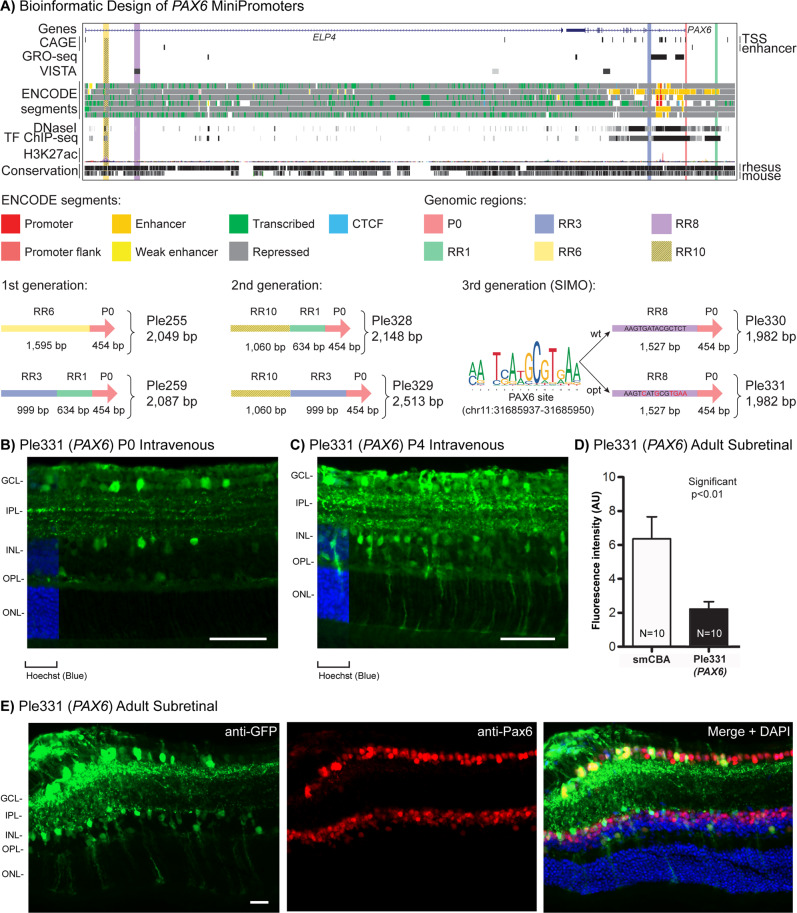
Development of *PAX6* MiniPromoters. **A** Bioinformatic design of first-generation Ple255 and Ple259, which were combined to produce the second-generation redesigns Ple328 and Ple329, also bioinformatic design of SIMO element Ple330 and base pair modified Ple331. ENCODE segments that potentially regulate the expression of the *PAX6* gene are highlighted horizontally. Vertically highlighted genomic regions correspond to their color-matched segments included in the MiniPromoter designs and are numbered as promoter(s) (P) and regulatory region(s) (RR). **B** Postnatal day 0 intravenous injection of Ple331-EmGFP, harvested 4 weeks later, led to robust expression with cell bodies in the ganglion cell layer (GCL), and the inner nuclear layer (INL). Scale bar, 100 µm. (For ubiquitous smCBA promoter see Fig. S1A). **C** Postnatal day 4 intravenous injection of Ple331-EmGFP, harvested 4 weeks later, led to robust expression with cell bodies in the GCL, INL, and processes that span the retina. Scale bar, 100 µm. (For smCBA see Fig. S1A.) **D** Adult subretinal injection of Ple331-EmGFP, harvested 4 weeks later, was quantified for epifluorescence intensity in RBPMS-positive ganglion cells, and showed significantly lower (~30%) ganglion cell expression when compared to smCBA (*p* < 0.01). (For smCBA see Fig. S1C.) **E** Adult subretinal injection of Ple331-EmGFP, harvested 4 weeks later, led to robust expression in the PAX6-positive cells, ganglion, amacrine, horizontal, and Müller glia, as indicated by co-staining with anti-PAX6. Scale bar, 20 µm. (For smCBA see Fig. S1C.) EmGFP emerald green fluorescent protein, IPL inner plexiform layer, N number of cells counted, ONL outer nuclear layer, OPL outer plexiform layer, TF transcription factor, TSS transcription start site. Green, anti-GFP; blue, Hoechst; red, anti-Pax6; yellow, merge.

We have previously demonstrated that first-generation MiniPromoters Ple255 (*PAX6*, 2049 bp) and Ple259 (*PAX6*, 2087 bp) (Table 1 and Fig. 7A) each showed restricted expression in three of four PAX6-positive cell types (Ple255: ganglion, amacrine, and horizontal; Ple259: ganglion, amacrine, and Müller glia) by direct intravitreal injection of rAAV [94].

Second-generation MiniPromoters were designed based on the partial success of Ple255 (*PAX6*) and Ple259 (*PAX6*), and incorporated the RRs contained in those MiniPromoters but now in new combinations to capture expression in all four PAX6-positive retinal cell types in a single MiniPromoter: Ple328 (*PAX6*, 2148 bp) and Ple329 (*PAX6*, 2513 bp) (Table 1 and Fig. 7A). Ple328 contains RR1 from Ple259 and a cut down of RR6 from Ple255 (RR10), RR10 contains a region focused on DNase I hypersensitivity, histone acetylation, and conservation [81]. Ple329 contains RR3 from Ple259 and a cut down of RR6 from Ple255 (RR10). All *PAX6* MiniPromoters use the same Promoter region, P0, the main transcription start site according to FANTOM5 CAGE data [94].

*PAX6* SIMO MiniPromoters were new designs based on the *PAX6* SIMO element: Ple330 (*PAX6* SIMO, 1982 bp) and Ple331 (*PAX6* SIMO, 1982 bp) (Table 1 and Fig. 7A). The SIMO element is an ultra-conserved sequence (uc.325) [95] located 150 kb downstream of *PAX6*. The VISTA enhancer project predicted an enhancer at this location [96], and studies in mouse and zebrafish show that the element acts as a strong enhancer in developing ocular structures [97]. In addition, mutations in the SIMO element cause aniridia, and a PAX6 binding site important for the genes autoregulatory feedback loop, and critical for maintenance of gene expression, is located in the element [97]. The binding of Pax6 to the SIMO element in mouse lens and forebrain was also confirmed by chromatin immunoprecipitation sequencing (ChIP-seq) [98]. New design Ple330 contains the 1527 bp SIMO element (RR8) and the P0 promoter, whereas new design Ple331 is base pair modified from Ple330 to optimize the PAX6 binding site according to the binding motif found in JASPAR [99, 100].

By neonatal intravenous delivery at P0 (the optimal injection time point for ganglion, amacrine, and horizontal cells), Ple331 (*PAX6* SIMO) expression was robust and restricted to the GCL and INL, for the INL the morphology was indicative of horizontal cells (Fig. 7B). By neonatal intravenous delivery at P4 (the optimal injection time point for the Müller glia), Ple331 expression was robust and restricted to the GCL, INL, and cell bodies in the INL with processes that span the retina, morphology indicative of Müller glia (Fig. 7C). Expression was absent in the brain and spinal cord, and was observed at a low level in the heart and pancreas (including the islets), and at a moderate level in the liver (Fig. S2G). At P0 with an early harvest at P6 to capture EmGFP expression that could be lost due to the rapid turnover of the PAX6-positive epithelial cells [101], Ple331 expression was observed throughout the cornea, in the epithelial layer, as well as unexpectedly in the stromal and endothelial layers (Fig. S2G). At the P6 time point liver expression was higher than the standard 4-week time point.

In contrast, the redesigned MiniPromoters Ple328 (*PAX6*) and Ple329 (*PAX6*), and new design Ple330 (*PAX6* SIMO) delivered at P0 resulted in expression in the GCL and INL, in cells with morphology indicative of horizontal cells, and in the cornea; but at P4 did not result in any expression in Müller glia.

All four PAX6 MiniPromoters went on to be characterized in the adult mouse eye by both subretinal and intrastromal delivery. Subretinal delivery of Ple331 (*PAX6* SIMO) resulted in robust and restricted expression to the GCL and INL. In the INL, there was morphology indicative of horizontal cells, as well as cell bodies and processes that spanned the retina indicative of Müller glia (Fig. 7D, E). Subretinal delivery of Ple328 (*PAX6*), Ple329 (*PAX6*), and Ple330 (*PAX6* SIMO) resulted in expression in the GCL and INL, for the INL morphology was indicative of horizontal cells, but did not result in any expression indicative of Müller glia. Intrastromal delivery of Ple328, Ple329, Ple330, and Ple331 resulted in expression in the corneal stroma, consistent with injection method and neonatal data.

Quantification was done for the most robust-specific MiniPromoter Ple331 (*PAX6* SIMO) by adult direct subretinal injection in one PAX6-positive cell type: ganglion cells. Quantification of EmGFP epifluorescence intensity in individual RBPMS-positive ganglion cells transduced with the same dose of virus, showed that Ple331-driven expression was reduced by ~1/3× relative to the ubiquitous smCBA promoter (*p* < 0.01) (Fig. 7D). Co-labeling with anti-PAX6 not only showed expression in the PAX6-positive cells, but also confirmed the endogenous-like expression pattern of the MiniPromoter (Fig. 7E).

## Discussion

### A resource of new, redesigned, and improved MiniPromoters for ocular gene therapy

We designed and tested 19 MiniPromoters to generate the final recommended seven. This resulting resource encompasses two new designs (Ple331 (*PAX6* SIMO) PAX6-positive cells, Ple348 (*PDE6H*) cone), two redesigns of previous MiniPromoters (Ple316 (*NR2E1*) Müller glia, Ple341 (*PCP2*) ON bipolar), and three improved characterizations of previous successful MiniPromoters (Ple32 (*CLDN5*) endothelial cells of the blood–retina/brain barriers, Ple253 (*PITX3*) corneal stroma, Ple265 (*PCP2*) ON bipolar). These recommended MiniPromoters range in size from 784 to 2484 bp. Although it may be possible to further cut down some of these human-gene MiniPromoters, it is also possible that they have reached their minimum functional size. At the current sizes, only a subset would be useful in self-complementary vectors, but all will be useful in single stranded (ss) rAAV, for therapies that involve small regulatory RNA and DNA molecules. Furthermore, in ss rAAV without the WPRE, these MiniPromoters can drive expression of proteins ranging from 517 to 1084 amino acids, which represent 62.9–90.2% of human proteins (calculated using UniProt [102] with one protein per gene). Finally, these promoters may be valuable for use in other viruses and gene therapy delivery systems.

To directly compare our MiniPromoters with promoters in the literature, one would need to clone them into the same viral genome, prepared by the same source in the same capsid, and delivered head to head. Furthermore, it would be ideal to test multiple capsids, to determine the best promoter/capsid combination for the cell type and delivery method. Nonetheless, in Supplementary Table 1 we present previously published promoters by our group and the most clinically advanced work of others. As there is a need for more restricted promoters, of varying strength, in this work we have developed promoters for cell types with differing degrees of promoter availability, from initial transgenic mouse work through to clinical trials. For ON bipolar cells, the most advanced promoter is mGluR6, which has shown restricted expression in both mouse and marmoset using rAAV2 variants and intravitreal administration [103]. For the cones, three promoters (PR, PR1.7, CAR) have been fully developed and are currently in use in clinical trials for the treatment of achromatopsia using rAAV2 variants, or rAAV8 and subretinal administration [10, 11, 104]. For the cornea, although multiple gene therapy avenues are being explored, currently the focus is on strong ubiquitous promoters [105], and not those specific for the cornea as we have done here. For the BRB and BBB, we are the primary contributor to the resource of promoters tested in rAAV [54, 55], and now recommend Ple32 (*CLDN5*). For the Müller glia, a promoter that expresses in both the Müller glia and retinal pigment epithelium (*RLBP1*) has been fully developed and is currently in use in a clinical trial for the treatment of *RLBP1 retinitis pigmentosa* using rAAV8 and subretinal administration [8, 104]; whereas Ple316 (*NR2E1*) resulted expression in the Müller glia with off-target expression in amacrine cells using rAAV9 and intravitreal or intravenous administration. For the PAX6-positive cells, we are the only group who have previously contributed to this resource [94], and now recommend Ple331 (*PAX6*).

### Not all cell-type restricted promoters in rAAV are weak

Quantification was undertaken to discover the relative strength of the leading recommended MiniPromoter per cell type by scoring the intensity of EmGFP epifluorescent driven in only the target cell type, compared to that of the ubiquitous smCBA promoter in that same cell type. Results ranged from two that were significantly stronger (Ple349 (*PDE6H*) ~4×, Ple32 (*CLDN5*) ~2×), one not significantly different (Ple316 (*NR2E1*) ~1×), to three significantly weaker (Ple265 (*PCP2*) ~1/2×, Ple331 (*PAX6* SIMO) ~1/3×, Ple253 (*PITX3*) ~1/5×). This is the first substantial data set showing that cell-type restricted promoters can be equal to, and even stronger than ubiquitous promoters. For clinical applications, it is important to note that quantification in mouse may not be sufficient to predict expression levels in primate [48].

### Controlled expression for intravenous gene therapy

The blood stream is the delivery approach, used either directly or indirectly, for most human therapeutics (small molecules and biologicals). The recent stunning success of the intravenously delivered rAAV9-augmentation gene therapy to children for spinal muscular atrophy [25, 26] helped propel this approach forward to clinical trials for several devastating incurable diseases (www.clinicaltrials.gov). However, this success was recently followed by the tragic death of two children with X-lined myotubular myopathy receiving intravenous rAAV8 therapy [106]. We do not yet know the mechanism of toxicity in these cases, but host variables, liver involvement, high viral dose, and features of vector design such as capsid and promoter may all have a role.

When considering the intravenous approach for targeting the eye, there are advantages and disadvantages. This approach may be particularly beneficial when the eye is fragile and less tolerant to direct-to-eye delivery (e.g. choroideremia [107]), or when the phenotypes go beyond the eye (e.g. aniridia [108, 109], Usher [110, 111], and Kearns-Sayre syndromes [112]). However, when delivering intravenously, or even direct to the eye where virus has the potential to escape into the blood stream, it may be important for safety and efficacy to consider not only the local off-target expression in the eye, but also the off-target expression body wide, with special focus on the liver. Therefore, we examined all MiniPromoters for off-target expression using rAAV9 intravenously in a developmental context.

MiniPromoters that displayed local off-target expression were set aside and not studied further. MiniPromoters that display off-target expression in other areas of the body that are expected based on the endogenous expression of the gene they were designed from, may have the potential to be further refined bioinformatically. This is exemplified by our work developing a MiniPromoter for the retinal ON bipolar cells from the gene *PCP2*, where expression in the cerebellum of the brain is endogenous and therefore expected, but in this context unwanted. Our original design Ple155 captured both expressions but through bioinformatics we were able to develop MiniPromoters Ple265 and 341 which maintained the ON bipolar expression but eliminated the cerebellum expression. MiniPromoters that display off-target expression in areas of the body that are not expected based on the endogenous gene may still be of value, but the ability to tackle this non-endogenous expression bioinformatically is very limited.

Finally, particularly in relation to toxicity it is worth considering the off-target liver expression we have observed for all MiniPromoters tested by intravenous delivery in this and our previous study [48]. Of the nine MiniPromoters tested in this study, none have expected strong endogenous liver expression. Yet at an early ~1-week time point, all displayed higher liver expression than at our standard 4-week time point. In contrast, this 1-week time point is when the central nervous system tissues we have examined would typically be low and increasing in expression toward 4 weeks. Thus, we find this liver expression to be uniquely promoter independent and decreasing with time.

### Pros and cons of using the therapeutic gene *PAX6*

When first contemplated, it may seem that for a genetic disorder, the promoter of the mutant gene is ideal to recapitulate the endogenous expression needed for the gene therapy to treat the disease. However, the disease state may result in the disruption of the development or cellular program regulating the mutant gene [6]. Thus, when the therapy is delivered, expression from that promoter no longer resembles the desired pattern. Even the expression of promoters from non-mutated genes may be abnormally regulated due to transcription factor changes in the disease environment. Thus, it may be necessary to step far away from the disease “pathway” to find a promoter with predictable, desirable, therapeutic expression.

Nevertheless, are there situations in which the disease gene may be the best promoter to drive therapy? Perhaps development or cellular pathways are “stuck”, and the gene therapy is able to reinitiate them and reestablish correct expression from the promoter. One might envision this for a disorder where, for example, reactivated stem/progenitor cells may normalize their expression, or where no protein is lost such as haploinsufficiency. Aniridia is such a disorder, in which haploinsufficiency of *PAX6* (a stem/progenitor cell gene) results in progression to blindness unhalted by current treatments [42]. In pursuing an endogenous-like promoter for this disorder, we were pleased with two new MiniPromoters: Ple330 (*PAX6* SIMO), and recommended Ple331 (*PAX6* SIMO). They are identical except the latter is base pair modified to optimize the PAX6 binding site according to the binding motif found in JASPAR [99, 100]. The unmodified form, Ple330, resulted in expression detected in three of the four PAX6-positive cells of the adult retina (ganglion, amacrine, and horizontal), but only the optimization Ple331 resulted in detection of expression in the weakest expressing of these cell types (Müller glia) [113, 114]. Even here, pitfalls abound when translating this promoter to therapy. In the haploinsufficient environment, will even the optimized Ple331 drive sufficient expression? On the other hand, might a self-regulation loop be created with PAX6 continuously driving the MiniPromoter generating PAX6. In fact, in such a situation might the weaker, and not recommended Ple330, be the better choice. At this point, promoter development leaves the realm of rationale design and enters the province of empirical results.

## Supplementary information

Supplementary Material
