# Supplementary material for: Human MiniPromoters for ocular-rAAV expression in ON bipolar, cone, corneal, endothelial, Müller glial, and PAX6 cells
Source: Gene Ther. 2021 Feb 2;28(6):351–72. doi: 10.1038/s41434-021-00227-z (PMC8222000; doi:10.1038/s41434-021-00227-z)
Supplement: Supplementary file 1 — Supplementary Material [file 41434_2021_227_MOESM1_ESM.docx]

| **Cell Type** | **Promoter** | **Delivery Method** | **Species** | **Reference PMID** | **Clinical Trial ID** |
| --- | --- | --- | --- | --- | --- |
| ON Bipolar | Ple155 (*PCP2*) | Transgenic, rAAV | Mouse | 24761428, 30765420, 26310623, 27164903 |  |
| ON Bipolar | Ple265 (*PCP2*) | Transgenic | Mouse | 30765420 |  |
| ON Bipolar | m*GluR6* | rAAV | Mouse, Marmoset | 27115727 |  |
| Cones | *IRBPe/GNAT2* | rAAV | Mouse, Dog | 24664760, 27052928 |  |
| Cones | *synGNAT2/GNAT2* | rAAV | Mouse | 24664760 |  |
| Cones | *PR1.7* | rAAV | Human | 29259520, 28391961 | NCT02935517, NCT02599922 |
| Cones | *hCAR* | rAAV | Human | 29259520, 28391961 | NCT03001310, NCT02946879 |
| Cones | *PR* | rAAV | Human | 29259520, 28391961 | NCT02610582 |
| Cornea | Ple67 (*FEV*) | rAAV | Mouse | 27164903 |  |
| Cornea | Ple251 (*C8ORF46*) | rAAV | Mouse | 27164903 |  |
| Cornea | Ple253 (*PITX3*) | rAAV | Mouse | 27164903 |  |
| Cornea | Ple302 (*DCX*) | rAAV | Mouse | 27164903 |  |
| Cornea | Ple303 (*NOV*) | rAAV | Mouse | 27164903 |  |
| Cornea | Kera | Transgenic | Mouse | 17001198 |  |
| BRB | Ple34 (*CLDN5*) | rAAV | Mouse | 27164903 |  |
| BRB | Ple261 (*CLDN5*) | rAAV | Mouse | 27164903 |  |
| BRB | *Tie-1* | Transgenic | Mouse | 15838304 |  |
| BRB | *Tie2* | Transgenic | Rat | 15948028 |  |
| Müller Glia | Ple264 (*NR2E1*) | Transgenic, rAAV | Mouse | 30765420, 27164903 |  |
| Müller Glia | *GFAP* | rAAV | Mouse, Rat | 20808778, 32194850, 28941260 |  |
| Müller Glia | *RLBP1* | rAAV | Mouse, NHP, Human | 26199951, 29359172, 30345694 | NCT03374657 |
| *PAX6* | Ple254 (*PAX6*) | rAAV | Mouse | 27556059 |  |
| *PAX6* | Ple255 (*PAX6*) | rAAV | Mouse | 27556059 |  |
| *PAX6* | Ple256 (*PAX6*) | rAAV | Mouse | 27556059 |  |
| *PAX6* | Ple257 (*PAX6*) | rAAV | Mouse | 27556059 |  |
| *PAX6* | Ple258 (*PAX6*) | rAAV | Mouse | 27556059 |  |
| *PAX6* | Ple259 (*PAX6*) | rAAV | Mouse | 27556059 |  |
| *PAX6* | Ple260 (*PAX6*) | rAAV | Mouse | 27556059 |  |

**Supplementary Table 1. Summary of previously published promoter studies from our group and the most clinically advanced studies of other groups.**

BRB, Blood Retina Barrier; NCT, clinical trial identifier (<https://clinicaltrials.gov>); PMID, PubMed ID (<https://pubmed.ncbi.nlm.nih.gov/>).

###
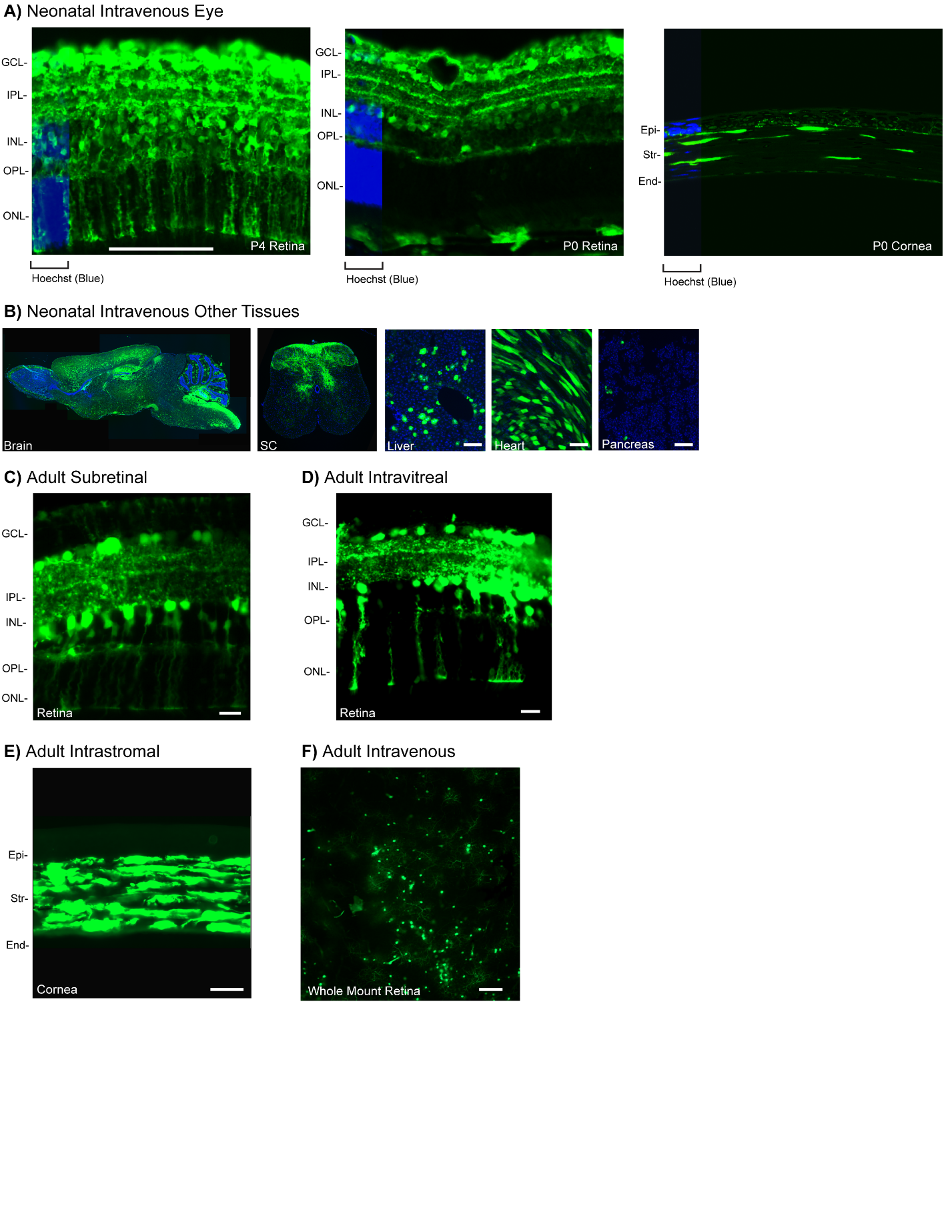


### Figure S1. The smCBA Promoter Showed Ubiquitous Expression.

**A)** Postnatal day (P) 4 intravenous injection of smCBA-EmGFP, harvested four weeks later, led to robust expression throughout the ganglion cell layer (GCL) and inner nuclear layer (INL). Postnatal day 0 intravenous injection of smCBA-EmGFP, harvested four weeks later, led to robust expression throughout the GCL and INL, as well as expression in the stromal (Str) and endothelial (End) layers of the cornea. Scale bar, 100 µm. **B)** Postnatal day 4 intravenous injection of smCBA-EmGFP, harvested four weeks later, led to robust ubiquitous expression in the brain, spinal cord (SC), liver, and heart, but weak expression in the pancreas. Scale bar, 100 µm. **C)** Adult subretinal injection of smCBA-EmGFP, harvested four weeks later, led to robust expression throughout the GCL and INL**.** Scale bar, 20 µm. **D)** Adult intravitreal injection of smCBA-EmGFP, harvested four weeks later, led to robust expression throughout the GCL and INL. Scale bar, 20 µm. **E)** Adult intrastromal injection of smCBA-EmGFP, harvested four weeks later, led to robust expression throughout the Str layer of the cornea. Scale bar, 50 µm. **F)** Adult intravenous injection of smCBA-EmGFP, harvested four weeks later and visualized by whole mount retina, led to robust expression. Scale bar, 100 µm. EmGFP, emerald green fluorescent protein; Epi, epithelial; IPL, inner plexiform layer; ONL, outer nuclear layer; OPL, outer plexiform layer. Green, anti-GFP; blue, Hoechst.

###
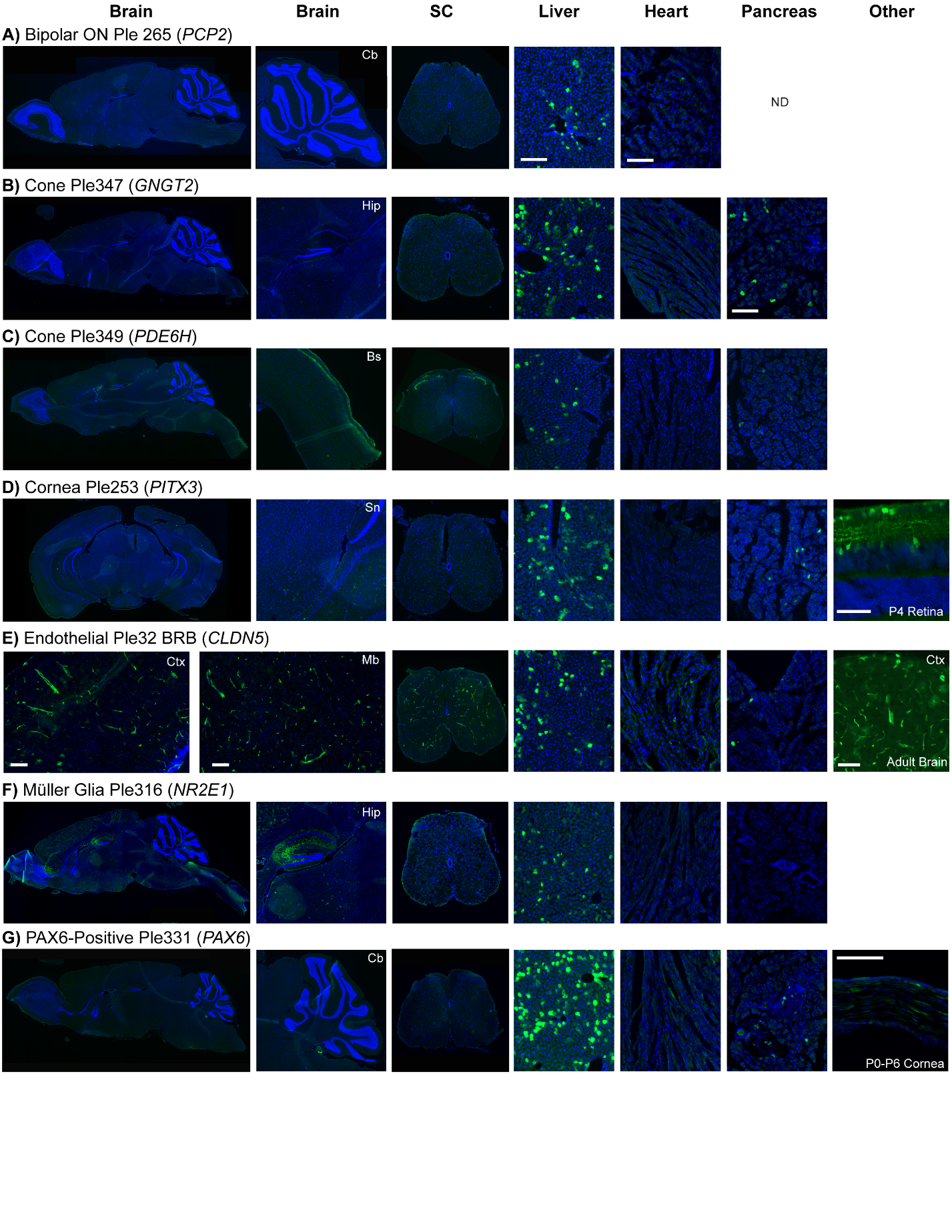


**Figure S2. Tissue Expression Patterns of MiniPromoters Delivered by Intravenous Injection in Neonatal Mice.**

**A)** Postnatal day (P) 4 intravenous injection of ON bipolar enriched Ple265-EmGFP (*PCP2*), harvested four weeks later, led to an absence of expression in the brain (including the cerebellum (Cb)), spinal cord (SC), and heart, but expression in the liver. **B)** P4 intravenous injection of cone enriched Ple347-EmGFP (*GNGT2*), harvested four weeks later, led to an absence of expression in the brain (including the hippocampus (Hip)), SC, and heart, but expression in the liver, and pancreas. **C)** P4 intravenous injection of cone enriched Ple349-EmGFP (*PDE6H*), harvested four weeks later, led to an absence of expression in the heart, and brain, but expression in the brain stem (Bs), dorsal SC, liver, and pancreas. **D)** P4 intravenous injection of cornea enriched Ple253-EmGFP (*PITX3*), harvested four weeks later, led to an absence of expression in the brain (including the substantia nigra (Sn)), SC, and heart, but expression in the liver, pancreas, and the GCL and inner nuclear layer of the retina. **E)** P4 intravenous injection of endothelial BRB enriched Ple32-EmGFP (*CLDN5*), harvested one week later, led to expression in the blood vessels throughout the brain, shown are the cortex (Ctx) and midbrain (Mb), as well as blood vessels of the spinal cord. P4 intravenous injection of Ple32-EmGFP, harvested four weeks later, led to expression in the liver, heart, and pancreas. Adult intravenous injection of Ple32-EmGFP, harvested four weeks later, led to expression in the endothelial cells of the blood-brain barrier. **F)** P4 intravenous injection of Müller glia enriched Ple316-EmGFP (*NR2E1*), harvested four weeks later, led to an absence of expression in the SC, heart, and pancreas, but led to expression in the brain (including the Hip), and liver. **G)** P4 intravenous injection of PAX6-positive enriched Ple331-EmGFP (PAX6), harvested four weeks later, led to an absence of expression in the brain (including the Cb), and SC, but expression in the liver, heart, and pancreas. P0 intravenous injection of Ple331-EmGFP, harvested six days later, led to expression throughout the cornea. Scale bars, 100 µm. BRB, blood retinal barrier; ND, not done. Green, anti-GFP; blue, Hoechst.
